# Gene Expression of Feed Intake-Regulating Peptides in the Gut–Brain Axis of Laying Hens Housed Under Two Different Egg Production Systems

**DOI:** 10.3390/ani15213127

**Published:** 2025-10-29

**Authors:** Kelly Johanna Lozano-Villegas, María Paula Herrera-Sánchez, Iang Schroniltgen Rondón-Barragán, Roy Rodríguez-Hernández

**Affiliations:** 1 Poultry Research Group, Faculty of Veterinary Medicine, Universidad del Tolima, Altos de Santa Helena, A.A 546, Ibagué 730006299, Colombia; kjlozanov@ut.edu.co (K.J.L.-V.); mpherreras@ut.edu.co (M.P.H.-S.); isrondon@ut.edu.co (I.S.R.-B.); 2 Immunobiology and Pathogenesis Research Group, Faculty of Veterinary Medicine, Universidad del Tolima, Altos de Santa Helena, A.A 546, Ibagué 730006299, Colombia; 3 Colombian Association of Veterinarians and Zootechnicians Specialists in Poultry (AMEVEA), Bogotá 111321, Colombia

**Keywords:** laying hen, welfare, feed intake, gut-brain axis, hypothalamus, neuropeptide, signaling pathway

## Abstract

Intensive farming methods have improved productivity, but public concerns have arisen regarding the welfare of production animals, particularly laying hens, and consumers demand higher animal welfare standards in all animal production systems. This study evaluated the impact of conventional cage (CC) versus cage-free (CF) housing systems on the gene expression of some peptide hormones regulating food intake along the gut–brain axis in laying hens at 80 wks. Sixty thousand Hy-Line Brown hens were reared under commercial farm conditions until week 15. At 16 wks, hens were randomly assigned into two housing systems: CC (450 cm^2^/bird) and CF (1111 cm^2^/bird). At week 80, hypothalamic, proventricular, duodenal, jejunal, and ileal tissues were sampled from six hens per system for qPCR analysis. Relative mRNA transcript levels of peptide hormones involved in the regulation of food intake (*GHRL*, *Ghsr*, *NPY*, *AGRP*, *POMC*, *CCK*, *CART*, *CRH*, *MC4R*, *MC1R*, *MC5R*) were quantified by qPCR using the most stable reference genes. CC hens exhibited upregulation of duodenal anorexigenic genes (*POMC*, *CCK*, *CART*, *CRH*) and stress-related *MC4R*, while CF hens showed higher ileal expression of foraging-related *AGRP*. No differences were observed in orexigenic peptides (*GHRL/Ghsr*, *NPY*). These findings suggest that housing systems differentially modulate gut–brain axis signaling. Specifically, CC environments appear to upregulate satiety signals, whereas CF systems seem to enhance the expression of genes linked to foraging behavior.

## Simple Summary

This study aims to address public concerns about laying hens’ welfare and the influence of conventional cage and cage-free production systems on the differential expression of genes associated with appetite-regulating metabolism. This study compared the impacts of conventional cage (CC) and cage-free (CF) systems on laying hens, analyzing gut–brain axis gene expression at 80 weeks. CC hens showed increased expression of anorexigenic and stress-related genes (*POMC*, *CCK*, *CART*, *CRH*, *MC4R*), while CF hens had higher ileal expression of foraging-related *AGRP*. No differences were found in orexigenic peptides (*GHRL/Ghsr*, *NPY*). Results suggest CC housing elevates satiety signals, whereas CF promotes foraging behavior.

## 1. Introduction

The growing global demand for animal protein, driven by rapid population growth, necessitates a transformation of food production systems [1]. In this context, the poultry industry has emerged as a crucial provider of high-quality protein through meat and egg production [2]. Traditionally, the industry has relied on conventional cage (CC) systems, which remain dominant worldwide because of their proven efficiency in terms of space and cost management [3]. However, these systems impose significant constraints on animal welfare by restricting natural behaviors, which can negatively impact health and physiological functions, such as metabolic disorders, diseases, and negative mental states, such as frustration or stress [3,4]. Consequently, a pronounced transition toward cage-free (CF) systems is underway, driven by consumer and regulatory pressures that prioritize improved animal welfare and sustainable practices [5,6]. This transition, while beneficial in supporting the natural behaviors of laying hens, also introduces new challenges related to environmental management and nutritional efficiency, with potential effects on the neuroendocrine regulation of feed intake [6,7].

In avian species, feed intake is modulated by the action of signaling molecules, such as peptide hormones expressed along the gut–brain axis, which maintain energy homeostasis by modulating appetite, gastrointestinal motility, and nutrient metabolism [8,9,10,11,12,13]. These signaling molecules are expressed in the central and peripheral nervous systems, facilitating crosstalk between the nervous and other major body systems, and can be divided into orexigenic peptides, such as neuropeptide Y (NPY) and agouti-related peptide (AgRP), which stimulate appetite, and anorexigenic factors, including proopiomelanocortin (POMC), cocaine- and amphetamine-regulated transcript (CART), corticotropin-releasing hormone (CRH) in the hypothalamus, and gut hormones, such as cholecystokinin (CCK) and peptide YY in the chicken gastrointestinal tract, which promote satiety [14]. The hypothalamus serves as a central integrator of this system, processing external environmental cues (particularly stressors) and generating appropriate physiological responses that influence feeding behavior [6].

Chickens encounter various acute and chronic stressors inherent to housing systems that may jeopardize their welfare and health, significantly disrupting their neuroendocrine balance [15]. The hypothalamic–pituitary–adrenal (HPA) axis plays a crucial role in the physiological stress response, affecting both immediate survival strategies and long-term health and fitness outcomes in avian species to maintain homeostasis during stress [16,17]. However, environmental stressors, such as temperature fluctuations and high stocking density, can disrupt this endocrine regulation [18], thereby affecting productive performance, egg production, health, and overall profitability [8,9]. The housing environment is a particularly significant modulator of avian physiology, with conventional cage systems linked to increased oxidative stress and altered expression of lipid metabolism-related genes [19,20,21], where physiological changes can lead to systemic metabolic alterations that affect feed utilization efficiency [22,23,24]. Furthermore, oxidative stress may compromise, potentially through influences on feed palatability, digestive efficiency and nutrient absorption [25].

However, a knowledge gap persists regarding the mechanistic insight into stress–hormonal regulation interactions [26]. This impedes the advancement of precision feeding approaches designed to address the distinct metabolic requirements associated with each production system [27]. Therefore, this study aimed to evaluate the effects of housing-stress on the gene expression of key appetite-regulating peptide hormones along the gut–brain axis in laying hens.

## 2. Materials and Methods

### 2.1. Animals and Management

Samples used were obtained from a previous study conducted on a commercial poultry farm situated in Ibagué, Tolima, Colombia (02°52′59″–05°19′59″ N, 74°24′18″–76°06′23″ W; elevation: 1250 m; mean temperature: 25 °C). This region is located between the central and eastern mountain ranges of the Colombian Andes.

**Figure 1:**
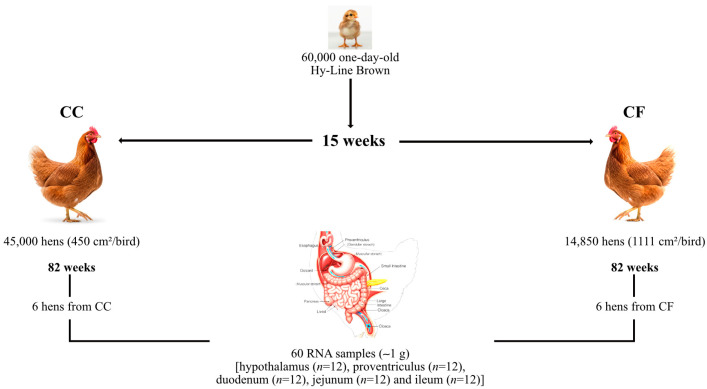
Experimental design for sample collection. Six hens from each housing system were selected to collect 60 tissue samples (~1 g) (hypothalamus (*n* = 12), proventriculus (*n* = 12), duodenum (*n* = 12), jejunum (*n* = 12), and ileum (*n* = 12)).

Under commercial conditions, 60,000 one-day-old Hy-Line Brown pullets were housed in manure-belt brood-grow cages (76.22 × 66.05 m) at a density of 16 pullets per cage (315 cm^2^/bird). The pullets were reared under standardized sanitary, nutritional, and management protocols according to the Hy-Line Brown Commercial Management Guide until they reached 15 weeks of age. Subsequently, they were transferred to two distinct housing systems, conventional cages (CC) and cage-free (CF), on the same farm and maintained until 82 weeks of age (Figure 1). In the CC system, 45,000 hens were housed in multi-tiered pyramidal (Californian-style) battery cages (40 × 45 × 40 cm). Each battery comprised four levels equipped with nipple drinkers, and the housing was ventilated using a cooling panel. The stocking density was four hens per cage (450 cm^2^/bird). For studies, 720 hens were evaluated in 15 replicates (12 cages per replicate, 48 hens per replicate).

The CF system consisted of an aviary design with deep-litter flooring (rice husks) in open-sided and naturally ventilated sheds. A total of 14,850 hens were distributed across two poultry houses at a density of 1111 cm^2^/bird. The houses were divided into 15 rooms (990 hens/room), which served as replicates for the CF system. Both systems followed identical lighting (14L:10D), feeding (Hy-Line Brown Layer Management Guidelines), and health protocols. Environmental conditions were comparable, with mean temperatures of 24.7 ± 2.81 °C (CC) and 24.45 ± 2.80 °C (CF) and a 7% humidity variation between the systems.

Both systems followed identical lighting, feeding, and health protocols. The lighting program followed the Hy-Line Brown Commercial Management Guide, using cool white LED fixtures with an intensity of 15–20 lux at bird head level, maintaining a 14L:10D photoperiod during the production phase. Environmental conditions were continuously monitored using automated data loggers, with mean temperatures of 24.7 ± 2.81 °C (CC) and 24.45 ± 2.80 °C (CF) and a 7% humidity variation between the systems, as reported in our companion study [28].

All procedures were approved in Act 007-2020 issued by the Bioethics Committee of the University of Tolima according to the Colombia Laws.

### 2.2. Sample Collection

At 80 weeks, six hens per system (CC and CF) were randomly selected from different replicates using a random sampling method without any pre-selection criteria. Hens were euthanized by cervical dislocation followed immediately by decapitation, consistent with AVMA guidelines. To ensure optimal RNA preservation, tissue samples were collected and immersed in RNAlater^®^ (Thermo Fisher Scientific, Waltham, MA, USA) within 5 min post-euthanasia. Hypothalamic, proventricular, duodenal, jejunal, and ileal tissue samples (~1 g) were collected, preserved in RNAlater^®^ (Thermo Fisher Scientific, Waltham, MA, USA), and stored at −20 °C.

### 2.3. Samples, RNA Extraction, cDNA Synthesis, and Endpoint PCR

Total RNA was extracted using TRIzol (Thermo Fisher Scientific), followed by treatment with DNase I (Promega, Madison, WI, USA). The integrity of the RNA was confirmed by agarose gel electrophoresis, and its purity was assessed spectrophotometrically, with an A260/A280 ratio greater than 1.8.

Complementary DNA (cDNA) was synthesized from 1 µg RNA using M-MLV reverse transcriptase (Promega). Endpoint PCR was performed to verify the quality of the cDNA by amplifying the *ACTB* gene. The reaction mixture (25 µL) comprised 14.8 µL of nuclease-free water, 5 µL of 5X GoTaq Flexi Buffer, 1 µL of dNTPs (1.5 mM; Invitrogen, Carlsbad, CA, USA), 1 µL of each primer (10 pmol/µL; Table 1), 1 µL of MgCl_2_ (25 mM), 0.125 µL of GoTaq Flexi DNA polymerase (Promega), and 1 µL of cDNA. Amplification was performed using the ProFlex PCR System (Applied Biosystems, Carlsbad, CA, USA) with an initial denaturation at 95 °C for 3 min, followed by 35 cycles of 95 °C for 30 s, 55 °C for 30 s, and 72 °C for 30 s, with a final extension at 72 °C for 5 min. The PCR products were electrophoresed on 2% agarose gels and stained with HydraGreen (ACTGene, Piscataway, NJ, USA).

### 2.4. Quantitative Polymerase Chain Reaction (qPCR)

Relative gene expression of reference and feeding regulation genes (Table 1) were measured using the Luna^®^ Universal qPCR Master Mix (New England BioLabs Inc., Beverly, MA, USA) in a QuantStudio 3 Real-Time PCR System (Thermo Fisher Scientific, Waltham, MA, USA), by fast ramp program, according to the manufacturer guidelines. No-template controls (NTCs) were included on every qPCR plate to monitor contamination. The thermal cycling conditions were as follows: denaturation for 1 min at 95 °C, 40 cycles of denaturation for 15 s at 95 °C, and annealing for 30 s at 60 °C. Subsequently, a melting step was performed at 95 °C for 1 s and 60 °C for 20 s, followed by a continuous increase in temperature to 95 °C at a rate of 0.15 °C/s. Each sample was analyzed in triplicate. Each biological replicate was analyzed in technical triplicate, and the resulting Cq values were averaged to yield a single value per biological replicate for subsequent statistical analysis. The specificity of amplification was confirmed by a single, sharp peak in the melting curve analysis for all genes.

### 2.5. Selection of Reference Genes

To assess the stability of the selected reference genes, data were analyzed using three different mathematical algorithms: geNorm [29], NormFinder [30], and BestKeeper [31]. The two most stable genes (ACTB and YWHAZ) were selected for normalization using the geometric mean of their expression values. Cycle quantification (Cq) values from triplicate qPCR runs were used for the analysis. All software packages were used according to the manufacturer’s instructions with default setting.

**Table 1:** Primers sequences used in the analysis of gene expression.

Type	Gene	Primer Sequences (5′–3′)		Product Size (bp)	Reference
***Feeding regulator***	*GHRL*	F	CTGGCTGGCTCTAGTTTTT	107	This study
		R	CAAAAGCTTTCTGTGCCT		
	*Ghsr*	F	TTTGTCCTCTTCTACCTGA	125	
		R	CTGGAGAGTCTTTTCTTTG		
	*NPY*	F	TGCTGACTTTCGCCTTGTCG	148	[16]
		R	GTGATGAGGTTGATGTAGTGCC		
	*AGRP*	F	CATCCTCACCTCGGACCTCA	163	
		R	CAGGGCCATCTGATCCAAGTCT		
	*POMC*	F	CGCTACGGCGGCTTCA	88	
		R	TCTTGTAGGCGCTTTTGACGAT		
	*CCK*	F	TTCTCTGTCCTAGGAAAC	168	
		R	GTACTCGTATTCTTCAGCAC		
	*CART*	F	CAGAGGTGCCGGTGTTGAG	140	
		R	TTCCCATAGCGAGCCCCCA		
	*CRH*	F	AGCAGCCCGATTTCTTCCCT	86	
		R	CAACAACTCGGCGGAGGCTT		
	*MC4R*	F	CAAGCGTGTAGGGGTCATCA	101	
		R	CAGATGATGACAACGCTGCTG		
	*MC1R*	F	GCCCTTCTTCTTCCACCTCAT	218	
		R	GCTCCGGAAGGCATAGATCA		
	*MC5R*	F	TCCATTCTTCCTCCATCTCATCC	157	
		R	CTTCCTCATTTCCTGGCTACG		
***Reference***	*GAPDH*	F	GAGGGTAGTGAAGGCTGCTG	113	[32]
		R	CATCAAAGGTGGAGGAATGG		
	*ACTB*	F	GCCCCCAAAGTTCTACAAT	110	
		R	AGGCGAGTAACTTCGTGTA		
	*18S*	F	CGAAAGCATTTGCCAAGAAT	98	
		R	GGCATCGTTTATGGTCGG		
	*YWHAZ*	F	AGGAGCCGAGCTGTCCAATG	85	
		R	CTCCAAGATGACCTACGGGCTC		
	*HMBS*	F	GGCTGGGAGAATCGCATAGG	131	
		R	TCCTGCAGGGCAGATACCAT		

### 2.6. mRNA Expression

The relative gene expression was determined by the 2^−ΔΔCt^ method [33], using the geometric mean of ACTB and YWHAZ genes for normalization. Values were expressed as fold change.

### 2.7. Statistical Analysis

The data were analyzed using descriptive analysis and the Shapiro–Wilk test. Additionally, the difference in gene expression was assessed using a parametric t-test for data with a normal distribution or a non-parametric Mann–Whitney test for data without a normal distribution, and the results are expressed as the mean ± SEM. The analyses were performed using GraphPad Prism v 10.0 (La Jolla, CA, USA), and the statistically significant differences were considered at *p* < 0.05.

### 2.8. Production Parameters Context

To contextualize the molecular findings within the birds’ overall physiological state, we reference key production data from our companion study [32] That work established that hens in the cage-free system, despite a significantly higher daily feed intake, had lower final body weights than their conventionally caged counterparts, while egg production remained comparable between the two groups.

## 3. Results

### 3.1. Reference Gene Expression Stability

Reference gene stability demonstrated significant variation across gastrointestinal tissues (Table 2). According to geNorm analysis, both *ACTB* (M = 1.0 in CF; 1.5 in CC–CF) and *YWHAZ* (M = 1.4 in CF; 1.5 in CC–CF) showed acceptable stability in the proventriculus. In the duodenum, both *YWHAZ* (M = 0.0 in CC; 0.5 in CF; 0.3 in CC–CF) and *GAPDH* (M = 0.0 in CC; 0.5 in CF; 0.3 in CC–CF) were identified as the most stable genes (Table 2). Similarly, in the ileum, *YWHAZ* and *GAPDH* exhibited consistent expression stability (M = 1.0 in CC; 1.1 in CF; 1.1 in CC–CF) (Table 1). In the jejunum, *YWHAZ* and *GAPDH* showed the highest stability (M = 0.8 in CC; 0.5 in CF; 0.6 in CC–CF) (Table 2).

Complementary BestKeeper analysis identified *ACTB* as the most stable gene in the proventriculus (SD = 0.7 in CC, 0.8 in CF, and 0.7 in CC–CF), showing consistent performance across all experimental conditions (Table 2). In the duodenum, *ACTB* also displayed stable expression (SD = 0.8 in CF), while in the ileum, *ACTB* maintained stability (SD = 0.8 in CC; 0.6 in CF) (Table 2).

According to NormFinder analysis, *ACTB* was consistently identified as the most stable reference gene across all gastrointestinal tissues, exhibiting the lowest stability values in the proventriculus (0.775 in CC; 0.516 in CF; 0.772 in CC–CF), duodenum (1.781 in CC; 1.084 in CF; 0.795 in CC–CF), ileum (0.753 in CC; 0.917 in CF; 0.811 in CC–CF), and jejunum (0.931 in CC; 0.931 in CF; 0.888 in CC–CF) (Table 3). Furthermore, the combination of *ACTB* and *YWHAZ* provided the highest overall stability in all tissues, with the lowest combined stability values ranging from 0.399 to 0.929 (Table 3).

### 3.2. Expression of Feed Intake Regulatory Peptides

**Figure 2:**
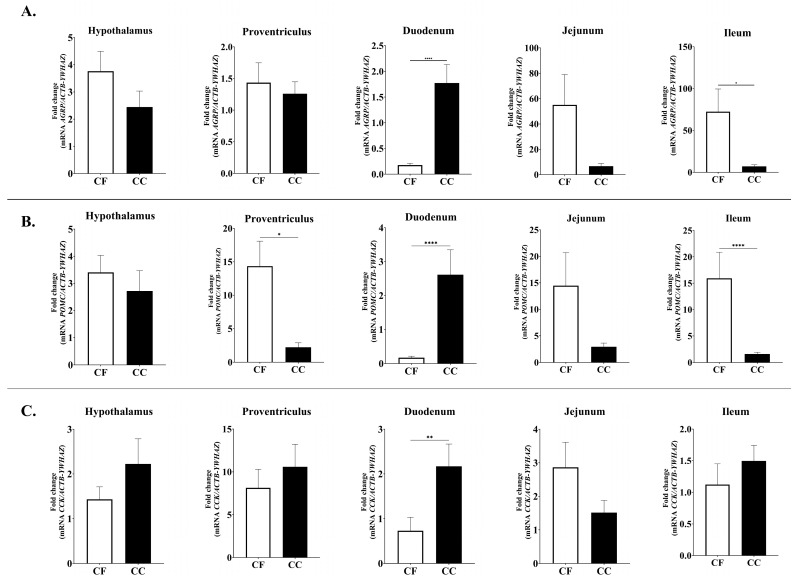
Relative mRNA expression of (**A**) *AGRP*, (**B**) *POMC*, and (**C**) *CCK* in the hypothalamus, proventriculus, duodenum, jejunum, and ileum; CC: conventional cage production system and CF: cage-free production system. The *ACTB* and *YWHAZ* genes were used as reference genes. Samples were run by triplicate throughout qRT-PCR. Data are presented as mean ± SEM. * *p* < 0.05, ** *p* < 0.01, **** *p* < 0.0001.

No significant differences were observed in the expression levels of orexigenic neuropeptides genes, including Ghrelin (*GHRL*), Ghrelin receptor (*GHSR*), and Neuropeptide Y (*NPY*). In contrast, the expression of agouti-related protein (*AGRP*) gene displayed significant spatial variation. Specifically, *AGRP* mRNA levels were markedly higher in the duodenum of CC hens compared with CF hens (*p* = 0.0001), whereas the ileum showed the opposite pattern (CF > CC, *p* = 0.0187) (Figure 2).

**Figure 3:**
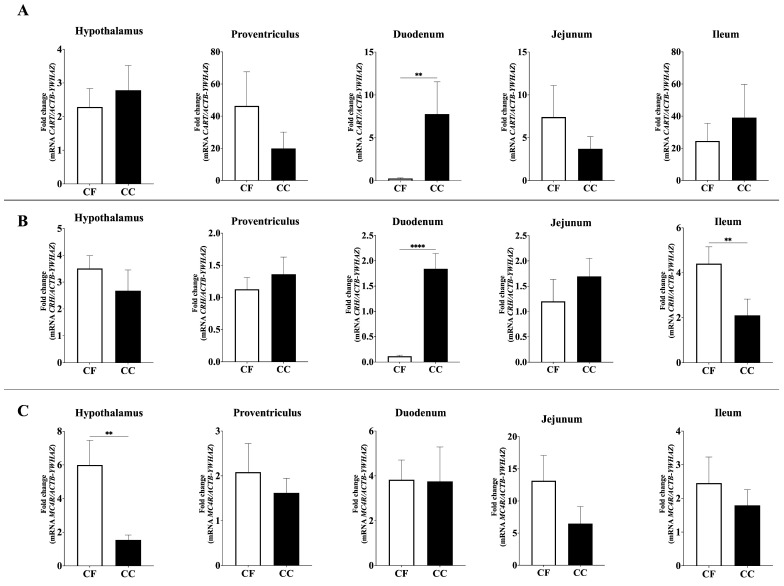
Relative mRNA expression of (**A**) *CART*, (**B**) *CRH*, and (**C**) *MC4R* in the hypothalamus, proventriculus, duodenum, jejunum, and ileum; CC: conventional cage production system and CF: cage-free production system. The *ACTB* and *YWHAZ* genes were used as reference genes. Samples were run by triplicate throughout qRT-PCR. Data are presented as mean ± SEM. ** *p* < 0.01, **** *p* < 0.0001.

Significant differences were also observed for all anorectic neuropeptide genes analyzed (Figure 2 and Figure 3). Duodenal proopiomelanocortin (*POMC*) mRNA expression was significantly higher in the CC group than in the CF group (*p* = 0.001), whereas *POMC* mRNA levels were elevated in the proventriculus and ileum in the CF group (*p* = 0.205, 0.001) (Figure 2). Similarly, duodenal Cholecystokinin (*CCK*; *p* = 0.0013) and Cocaine- and Amphetamine-Regulated Transcript (*CART*; *p* = 0.0016) expression levels were significantly higher in CC hens compared to CF hens (Figure 2 and Figure 3).

Furthermore, Corticotropin-Releasing Hormone (*CRH)* expression exhibited distinct regional regulation: duodenal *CRH* mRNA abundance was significantly upregulated in CC hens (*p* = 0.0001), whereas *CRH* expression in the ileum was higher in CF hens (*p* = 0.0033) (Figure 3). Finally, among the melanocortin receptor genes, Melanocortin 4 Receptor (*MC4R*) expression in the hypothalamus was significantly higher in hens housed under the CC system compared to those in CF (*p* = 0.0013), while no significant differences were observed in *MC5R* or *MC1R* expression levels.

## 4. Discussion

In the context of production environments, housing constitutes one of the most critical external factors influencing both the production and quality of eggs in laying hens [34]. Housing systems, such as CF and CC, have a significant impact on feed intake [35], which is particularly important given that the performance of laying hens (productivity and laying efficiency) mainly depends on effective feed management [23,36]. As reported by Dikmen et al. (2016), laying hens in cage-free systems, particularly those in free-range environments, exhibit higher feed consumption than their counterparts in conventional cages [37]. This increase is primarily associated with higher levels of physical activity, including foraging and locomotion, which elevates energy expenditure and consequently demands a higher feed intake to meet metabolic and nutritional requirements [37]. Similarly, Rodríguez-Hernández et al., 2024 demonstrated these impacts clearly: at 82 weeks of age, hens reared in cage-free systems displayed significantly higher feed intake, but lower body weight compared to those in conventional cages [28].

Avian feeding behavior is regulated by central and peripheral signaling, coordinated by the nervous, endocrine, and digestive systems through conserved mechanisms [8,38]. This regulatory system depends on specialized organs, such as the liver, pancreas, adipose tissues, gastrointestinal tract, and brain, which play crucial roles in nutrient sensing, energy homeostasis, and signal transduction related to hunger and satiety [39]. In poultry, the digestive system comprises the craw, proventriculus, gizzard, small intestine (duodenum, jejunum, and ileum), large intestine (including the cecum and rectum), and cloaca [40]. Each organ contributes uniquely to nutrient processing and signal generation, with the proventriculus serving as a source of ghrelin, the duodenum acting as the primary nutrient sensor and CCK release site, and the jejunum and ileum involved in the “ileal brake” and long-term satiety [41,42,43].

In this study, distinct gene expression profiles in appetite-regulating pathways were identified between CC and CF production systems, with significant differential expression observed in key neuropeptide and melanocortin genes. Peripheral signals are integrated by central nervous system structures, particularly hypothalamic and brainstem regions, which coordinate feeding responses based on metabolic status [44]. Among peripheral signals, feeding regulatory neuropeptides plays a pivotal role in maintaining energy homeostasis [45]. Genes encoding feeding regulatory neuropeptides through the production of orexigenic and anorexigenic signals are critical for the formation of a sensing and signaling network that regulates food intake [38,46]. Therefore, as these mechanisms play a crucial role in maintaining growth and productive efficiency [47], elucidating their impact on feeding behavior is imperative to advance commercial breeding practices and sustainability in the poultry industry [8].

Alterations in gene expression may be attributed to stress factors during housing conditions [32], and these stress-associated responses can include changes in the transcription of multiple genes related to metabolism, such as those encoding feed intake regulatory peptides [48]. Among orexigenic peptides, neuropeptides Y (NPY), agouti-related peptide (AGRP), and melanin-concentrating hormone (MCH) act centrally to stimulate appetite [49,50]. Particularly, AGRP plays a central role in energy balance by antagonizing melanocortin receptors, which stimulate feeding and decreasing energy expenditure [51]. In this study, the expression of *AGRP* mRNA in the duodenum was significantly higher in hens housed in the CC system compared to those in the CF system, which may be associated with stress induced by the restrictive conditions of CC system. As previously indicated, CC may generate stress responses [51], and stressful situation increase *AGRP* mRNA levels [52]. Conversely, ileal *AGRP* expression was significantly higher in the CF group than in the CC group, which may be attributed to the ability of hens in CF systems to express natural behaviors. As AGRP stimulates food-seeking and feeding behaviors, its overexpression could be linked to greater opportunities for active foraging and exploratory feeding [53,54].

Regarding anorexigenic neuropeptide (appetite-suppressing), significative differences were observed on *POMC* (Proopiomelanocortin), *CCK* (Cholecystokinin), *CART* (Cocaine- and amphetamine-regulated transcript), and *CRH* (Corticotropin-releasing hormone) gene expression. *POMC* is a polypeptide precursor of several peptides and hormones with a wide range of physiological actions [55]. In birds, *POMC* mRNA expression is primarily localized in the pituitary gland, brain regions, and hypothalamus, with the latter being the tissue where *POMC* exerts its primary function as a regulator of neuronal circuits associated with the control of feeding and energy metabolism [56,57]. However, studies have identified that *POMC* mRNA are present in many nonpituitary tissues such as the duodenum, kidney, colon, liver, lung, stomach, and spleen [58].

In this study, *POMC* mRNA expression in the duodenum was significantly higher in the CC group compared to the CF group, which is consistent with previous findings indicating that, among non-pituitary tissues, the duodenum exhibits relatively high concentrations of this peptide [58]. Also, the upregulation of the *POMC* mRNA may be associated with a stress-induced response, as elevated levels of this peptide have previously been linked to stress-related mechanisms [59,60]. Conversely, in the duodenum, *POMC* mRNA expression in the proventriculus and ileum was higher in hens from the CF group, which is significant given the crucial role of the proventriculus and ileum in nutrient chemosensing and absorption of satiety signaling [61]. In this case, the overregulation of *POMC* observed in the proventriculus and ileum of hens from the CF group may be attributed to increased energy expenditure resulting from enhanced physical activity and greater freedom of movement. Moreover, this finding is consistent with previous studies reporting higher POMC expression under conditions of elevated energy intake [62].

Concerning *CCK*, this peptide is known as a gastrointestinal hormone that controls feed intake in the short term (i.e., meal to meal) through a satiety signal to the brainstem capable of depressing appetite [44,63]. Nevertheless, *CCK* has multiple effects on the gastrointestinal system, including gallbladder contraction, gut motility, gastric emptying, and secretion of gastric acid and pancreatic enzymes [44,64]. In this study, *CCK* mRNA expression in the duodenum was significantly higher in the CC group than in the CF group. This is relevant because the duodenum is considered one of the principal organs that affect feed intake in chickens [65]. Similar to *CCK*, *CART* mRNA expression in the duodenum was significantly higher in the CC group than in the CF group. In this context, the mRNA expression level of *CART* may be associated with its role in the physiological stress response [66]. In this case, stress is likely induced by housing conditions, which may explain the upregulation of *CART* in hens in the CC group compared to those in the CF group. Additionally, *CRH* mRNA levels in the duodenum were higher in CC group, whereas in the ileum, *CRH* expression was higher in the CF group. Nevertheless, it is important to note that *CRH* mRNA expression is highly dependent on the nature of the stimulus, with reports indicating either an upregulation, downregulation, or no significant change [33].

As previously mentioned, central nervous system (CNS) plays a pivotal role in regulating appetite and energy balance in poultry by integrating and processing peripheral signals to coordinate appropriate responses [40]. Within the CNS, hypothalamic neural circuits have been recognized as a central processor that regulates food intake and energy balance through the central melanocortin system [40]. As in mammals, in avian central melanocortin system peptides exert their effects by acting on melanocortin receptors (MCRs) in the hypothalamus [67]. Also, among *MCRs*, *MC4R* plays a central role in the regulation of appetite and body weight homeostasis [68]. In this study, no significant differences were observed in the expression of *MC5R* and *MC1R*, whereas the expression of *MC4R* was significantly higher in hens housed in a CC environment than in those housed in a CF environment. The upregulation of *MC4R* may be linked to a more effective regulatory system that enhances energy use and controls appetite [69]. However, it is important to note that this gene expression is not specific to any particular genetic polymorphism (AA, AB, or BB genotypes) [69], which is relevant because several authors have reported that certain genotypes are significantly associated with higher body weight compared to others [70,71].

Numerous studies have identified reliable reference genes in laying hens; nevertheless, only a limited number have examined suitable reference genes under different egg production systems [32,72]. To date, no studies have reported reference genes for the proventriculus, duodenum, ileum, jejunum, or hypothalamus under different housing conditions. In this study, the expression stability of the five reference genes varied depending on the algorithm used. For example, *ACTB* was identified as the most stable reference gene according to BestKeeper and NormFinder, whereas YWHAZ was the most stable according to geNorm and NormFinder. The *ACTB* gene, which codes for beta-actin an abundant and highly conserved cytoskeleton structural protein [73], which is frequently employed as a validated reference gene in several [72,74,75,76], and also the most often used non-validated reference gene in chicken tissues [77]. Regarding *YWHAZ*, this gene encodes tyrosine 3-monooxygenase/tryptophan 5-monooxygenase activation protein, zeta polypeptide, which participates in diverse signaling pathways regulating cellular proliferation, migration, and differentiation [78,79]. Based on the stability patterns observed in this study, the combined use of *ACTB* and *YWHAZ* is recommended as a reliable reference gene.

## 5. Conclusions

In summary, this study provides a comprehensive analysis of mRNA expression changes in feed intake regulatory peptides across the hypothalamus, duodenum, ileum, proventriculus, and jejunum of laying hens reared in two distinct production systems. Our findings advance the understanding of how environmental conditions influence feed intake regulation, as differential gene expression patterns suggest that the housing system significantly impacts avian physiology. However, further research is warranted. Although alterations in mRNA expression are an important factor, the protein is the final biological product that ultimately induces physiological changes.

## Figures and Tables

**Table 2:** Stability values of reference genes, ranked by the geNorm and BestKeeper algorithms, for the proventriculus, duodenum, ileum, and jejunum of laying hens at 80 weeks of production.

Tissue	geNorm							BestKeeper						
		CC		CF		CC-CF			CC		CF		CC-CF	
		Ranking	M Value	Ranking	M Value	Ranking	M Value		Ranking	SD	Ranking	SD	Ranking	SD
**Proventriculus**	1	*18S*	1.8	*ACTB*	1.0	*ACTB*	1.5	1	*ACTB*	0.7	*ACTB*	0.8	*ACTB*	0.7
	2	*HMBS*	1.8	*HMBS*	1.0	*YWHAZ*	1.5	2	*YWHAZ*	1	*YWHAZ*	1	*YWHAZ*	1
	3	*ACTB*	2.4	*YWHAZ*	1.4	*18S*	2.5	3	*GAPDH*	2.5	*HMBS*	1	*18S*	2.5
	4	*YWHAZ*	2.8	*GAPDH*	2.1	*HMBS*	2.8	4	*18S*	2.6	*18S*	2.3	*GAPDH*	2.5
	5	*GAPDH*	3.5	*18S*	2.7	*GAPDH*	3.3	5	*HMBS*	3.1	*GAPDH*	2.6	*HMBS*	2.7
**Duodenum**	1	*YWHAZ*	0	*YWHAZ*	0.5	*YWHAZ*	0.3	1	*ACTB*	2.0	*ACTB*	0.8	*ACTB*	1.8
	2	*GAPDH*	0	*GAPDH*	0.5	*GAPDH*	0.3	2	*HMBS*	3.4	*18S*	1.6	*YWHAZ*	3.1
	3	*18S*	2.5	*ACTB*	1.9	*ACTB*	2.4	3	*YWHAZ*	3.7	*YWHAZ*	2.5	*GAPDH*	3.1
	4	*ACTB*	3.4	*18S*	2.8	*18S*	3.0	4	*GAPDH*	3.7	*GAPDH*	2.5	*18S*	3.2
	5	*HMBS*	4.5	*HMBS*	3.5	*HMBS*	3.9	5	*18S*	4.3	*HMBS*	3.1	*HMBS*	3.5
**Ileum**	1	*YWHAZ*	1.0	*YWHAZ*	1.1	*YWHAZ*	1.1	1	*ACTB*	0.8	*ACTB*	0.6	*ACTB*	1.0
	2	*GAPDH*	1.0	*GAPDH*	1.1	*GAPDH*	1.1	2	*18S*	0.8	*18S*	1.6	*18S*	1.6
	3	*ACTB*	1.6	*ACTB*	1.7	*ACTB*	1.6	3	*YWHAZ*	1.8	*YWHAZ*	1.8	*YWHAZ*	2
	4	*18S*	2.2	*18S*	2.5	*18S*	2.3	4	*HMBS*	2.3	*GAPDH*	2.3	*GAPDH*	2.6
	5	*HMBS*	2.7	*HMBS*	3.1	*HMBS*	2.8	5	*GAPDH*	2.8	*HMBS*	2.6	*HMBS*	2.8
**Jejunum**	1	*YWHAZ*	0.8	*YWHAZ*	0.5	*YWHAZ*	0.6	1	*ACTB*	1.5	*18S*	1	*18S*	1.4
	2	*GAPDH*	0.8	*GAPDH*	0.5	*GAPDH*	0.6	2	*18S*	0.1	*ACTB*	1.6	*ACTB*	1.5
	3	*ACTB*	1.7	*ACTB*	1.5	*ACTB*	1.5	3	*YWHAZ*	2.5	*HMBS*	2.8	*HMBS*	2.7
	4	*18S*	2.6	*18S*	2.3	*18S*	2.4	4	*HMBS*	2.6	*YWHAZ*	3.3	*YWHAZ*	2.9
	5	*HMBS*	3.2	*HMBS*	2.8	*HMBS*	2.9	5	*GAPDH*	2.8	*GAPDH*	3.6	*GAPDH*	3.3

SD: standard deviation; CC: conventional cage; CF: cage-free; CC-CF: overall data.

**Table 3:** Stability values of reference genes ranked by the NormFinder algorithm in the proventriculus, duodenum, ileum, and jejunum of laying hens at 80 weeks of production.

Tissue	NormFinder								
		CC		CF		CC-CF		The Best Combination of Two Genes	
		Ranking	Stability Value	Ranking	Stability Value	Ranking	Stability Value	Genes	Stability Value
**Proventriculus**	1	*ACTB*	0.775	*ACTB*	0.516	*ACTB*	0.772	*ACTB-YWHAZ*	0.399
	2	*YWHAZ*	1.108	*HMBS*	0.516	*YWHAZ*	1.016		
	3	*18S*	2.604	*YWHAZ*	0.532	*HMBS*	2.925		
	4	*HMBS*	3.476	*GAPDH*	3.505	*18S*	2.961		
	5	*GAPDH*	4.32	*18S*	3.58	*GAPDH*	3.743		
**Duodenum**	1	*ACTB*	1.781	*ACTB*	1.084	*ACTB*	0.795	*ACTB-YWHAZ*	0.838
	2	*18s*	2.205	*18S*	1.929	*18S*	1.983		
	3	*YWHAZ*	3.184	*GAPDH*	3.055	*GAPDH*	2.98		
	4	*GAPDH*	3.184	*YWHAZ*	3.077	*YWHAZ*	2.984		
	5	*HMBS*	5.82	*HMBS*	4.3	*HMBS*	4.878		
**Ileum**	1	*ACTB*	0.753	*ACTB*	0.917	*ACTB*	0.811	*ACTB-YWHAZ*	0.537
	2	*YWHAZ*	1.576	*18S*	0.1886	*18S*	1.751		
	3	*18S*	1.803	*YWHAZ*	2.248	*YWHAZ*	1.841		
	4	*GAPDH*	2.723	*GAPDH*	3.004	*GAPDH*	2.766		
	5	*HMBS*	3.212	*HMBS*	3.7	*HMBS*	3.384		
**Jejunum**	1	*ACTB*	0.931	*ACTB*	0.931	*ACTB*	0.888	*ACTB-YWHAZ*	0.929
	2	*YWHAZ*	2.192	*YWHAZ*	1.994	*YWHAZ*	2.026		
	3	*18S*	2.33	*18S*	2.071	*18S*	2.1		
	4	*GAPDH*	3.006	*GAPDH*	2.481	*GAPDH*	2.634		
	5	*HMBS*	3.738	*HMBS*	3.295	*HMBS*	3.474		

CC: conventional cage; CF: cage-free; CC-CF: overall data.

## Data Availability

Data are contained within the article.

## Abbreviations

The following abbreviations are used in this manuscript:

CC	Conventional Cage
CF	Cage Free
